# Development of a training-oriented wearable knee joint exoskeleton for forming a scientific force application pattern in squat tasks

**DOI:** 10.3389/fbioe.2026.1719023

**Published:** 2026-02-13

**Authors:** Shuai Chang, Feng Feng, Zihao Li, Yang Yu

**Affiliations:** 1 Sport Biomechanics Center, Institute of Artificial Intelligence in Sports, Capital University of Physical Education and Sports, Beijing, China; 2 Department of Physical Education, Capital Normal University, Beijing, China

**Keywords:** injury prevention, joint power redistribution, muscle activation, squat biomechanics, wearable exoskeleton

## Abstract

**Background:**

Squat training enhances athletic performance but poses knee injury risks when the technique is poor.

**Objective:**

Develop a resistance-type wearable knee exoskeleton to cultivate a hip-dominant, knee-safe squat pattern.

**Methods:**

Fifteen healthy men performed squats with either the exoskeleton or with barbells at three matched loads. Three-dimensional motion, ground-reaction force, and electromyography data were processed in OpenSim and MATLAB to quantify joint kinematics, power share, and muscle contribution.

**Results:**

The exoskeleton significantly reduced average angular velocity at the hip, knee, and ankle (p < 0.05), increasing hip power contribution by 20%–40% while decreasing knee contribution by 20%–30%, confirming a hip-driven pattern. However, knee and ankle ranges of motion decreased by 7°–9°, and vastus medialis activation dropped by ∼50% (p < 0.05).

**Conclusion:**

The device effectively standardizes squat mechanics and off-loads the knee, yet individualized tuning and auxiliary mobility work are recommended to optimize training transfer and preserve functional range of motion.

## 1 Introduction

As a fundamental movement in strength training and functional rehabilitation, squats are also widely used to improve athletic performance and daily functional movements. They effectively enhance the strength and stability of the lower limbs and core muscle groups (Salem et al., 2003). Research indicates that the squat movement mimics basic actions in daily life, such as standing up, sitting down, and lifting objects, having a significant role in improving lower limb explosive power, enhancing neuromuscular control, and boosting proprioception (Schoenfeld, 2010; Escamilla, 2001). However, the biomechanical risks associated with the squat movement are closely related to the technique used in its execution. Current biomechanical analyses emphasize adopting a hip-dominant force application pattern that fully activates the gluteal muscles and proximal hip muscles to distribute lower limb loads and reduce knee stress (Strau and Powers, 2024). This “hip-dominant” strategy effectively reduces the anterior knee joint shift and patellofemoral pressure by increasing hip flexion and posterior displacement, which is crucial for preventing knee injuries. However, improper squat technique, such as excessive knee valgus, anterior knee shift, or insufficient activation of hip muscles, can cause excessive stress on knee structures (including the anterior cruciate ligament and patellofemoral cartilage), significantly increasing the risk of sports injuries (Myer et al., 2014). Electromyography and kinematic studies have also found that insufficient activation of the hip abductors and external rotators can lead to poor force lines in the lower limbs, which is highly correlated with chronic knee pain and injury (Ayotte et al., 2007). Scholars have pointed out that “to protect the knee joint, squats should emphasize hip flexion and posterior displacement, reducing anterior knee movement and patellofemoral stress” (Lorenzetti et al., 2012). Therefore, preventing knee injuries during training and rehabilitation is important, and forming a scientifically guided hip-dominant force application pattern is particularly crucial. Helping practitioners realize standardized movement patterns and appropriate force lines has become an important future direction for sports protection and performance enhancement.

In the field of injury rehabilitation, systematic and targeted movement training is recognized as a core method of promoting the recovery of neuromuscular function, improving joint range of motion, and enhancing patients’ quality of life (Pollock et al., 2013). As the number of patients with various neurological injuries, musculoskeletal disorders, and chronic degenerative diseases increases yearly, traditional physical therapy and manual training have been limited by treatment intensity, time, and professional manpower investment and cannot fully meet the growing rehabilitation needs. In recent years, robotic and exoskeleton technologies have gradually found applications in rehabilitation medicine, particularly showing promising prospects for the restoration of motor function in patients with stroke, spinal cord injuries, and neurodegenerative diseases (Reinkensmeyer et al., 2004). These systems can provide patients with high-intensity, repetitive, and programmable movement training, effectively stimulating brain plasticity and promoting neural pathway reformation while greatly reducing the physical burden on therapists and improving training efficiency (Lo et al., 2010). Compared to traditional rehabilitation training, robotic and exoskeleton-assisted systems have several significant advantages (Prange et al., 2006). First, these devices can precisely control joint motion trajectories, force output, and training parameters, enabling highly personalized and objectively quantifiable rehabilitation training. Second, robotic and exoskeleton systems can adjust assistance or resistance levels in real time based on the patient’s rehabilitation progress, facilitating progressive training, encouraging more active patient engagement, and accelerating functional recovery. Intelligent feedback and data collection capabilities can assess movement performance and progress in real time, providing data support for clinical decision-making and the formulation of precise treatment plans (Strau and Powers, 2024; Reinkensmeyer et al., 2004). Existing studies have shown that assistive robots and exoskeleton training can significantly enhance patients’ limb mobility and daily self-care abilities and have a positive impact on the rehabilitation of upper and lower limb functions. This also provides a solid technical foundation for the development of scientific force application patterns in complex movements, such as squats, and the prevention of sports injuries (Ding et al., 2018).

In the fields of sports medicine and occupational health, an increasing number of studies and industry practices have shown that proactive prevention before injuries occur is more effective than post-injury interventions, significantly reducing injury incidence and improving overall health levels and work efficiency (Singer and Donoso, 2009). Preventive measures, including scientific guidance on movement patterns, reasonable physical training, optimization of force lines, and safety education, can help reduce chronic injuries to soft tissues, joints, and bones (Moser and Schatz, 2012). On this basis, in recent years, assistive exoskeleton systems have been developed and widely applied in various fields such as industry, healthcare, rehabilitation, and military to address the injury risks posed by repetitive bending and stretching, heavy lifting, and prolonged abnormal postures in sports and occupational environments (De Looze et al., 2016). Assistive exoskeletons can effectively reduce local loads by providing mechanical support or active assistance to key joints and muscle groups, controlling the biomechanical characteristics during high-risk movements, and minimizing the probability of injuries caused by excessive force, improper posture, and cumulative fatigue (Huysamen et al., 2018). Numerous ergonomic and biomechanical assessments have confirmed that both electrically assisted exoskeletons and flexible wearable assistive systems offer advantages in reducing peak muscle activation, decreasing loads on the lumbar and lower limb joints, and alleviating occupational fatigue (Jo and Bae, 2017). Additionally, exoskeletons offer new means for biomechanical control and movement standardization in high-load, high-repetition movements, such as squats, lifting, and maintaining standing positions, providing solid technical support for preventing sports and occupational injuries, optimizing force application patterns, and enhancing training and operational safety (Golabchi et al., 2022).

Although exoskeletons may help prevent knee injuries and enhance athletic performance, the long-term effects of such devices on muscular adaptation and autonomous motor control remain unclear. Prolonged or inappropriate reliance on external support may reduce intrinsic activation of key muscle groups, alter neuromuscular coordination patterns, and potentially impair athletes’ ability to independently control complex movements. Therefore, how to balance external support with intrinsic muscular activation, and how to use exoskeletons as a temporary guidance tool rather than a permanent substitute for active control, have become important scientific and practical questions in the application of exoskeletons to sports training.

At the same time, significant individual differences in squat technique exist, with each athlete possessing distinct technical characteristics and biomechanical patterns. Variations in hip–knee coordination, center-of-mass distribution, ankle mobility, and muscle activation timing may influence both the extent to which an individual benefits from external guidance and how the exoskeleton affects joint loading and force distribution. These individual differences may, in turn, affect the applicability, training efficacy, and safety of the same exoskeleton device across users and call for exoskeleton designs that allow adjustable resistance profiles and personalized configuration to accommodate diverse movement techniques.

Currently, various technical solutions for squat training or knee joint protection include resistance training devices using elastic bands, passive knee support braces, and some commercial exoskeleton devices (Ranaweera et al., 2018; Shamaei et al., 2014). Elastic band devices can provide basic resistance but cannot precisely control the timing and magnitude of force output and are difficult to dynamically adjust according to individual needs. Passive support braces mainly limit abnormal joint motion and lack active guidance functions for force application patterns. Some existing exoskeleton devices suffer from issues such as complex structures, inconvenient wearing, or non-linear resistance adjustment (Chen et al., 2016). For example, knee joint exoskeletons based on damping rotating shafts can provide a certain level of torque resistance, but the tension of the adjustment nut has a non-linear relationship with the output friction torque, and the adjustment range of the adjustment nut is limited, leading to difficulties in precise torque regulation. Furthermore, the fastening components cannot withstand vertical forces, which may cause the exoskeleton to slip downward (Duvivier, 2021). In addition, most existing exoskeleton systems for squat or lower limb training focus on joint protection or load sharing and rarely provide targeted guidance on hip-dominant force application patterns; they also do not sufficiently consider how to monitor or regulate the balance between external support and intrinsic muscle activation over repeated training sessions.

Compared with exoskeleton devices reported in similar studies, the device developed in this study has several distinctive features and is specifically designed to address the above limitations. First, it adopts a clutch disc structure rather than a traditional damping rotating shaft to achieve stepless and precise torque adjustment, solving the problem of imprecise torque regulation in existing devices. Second, the fastening mechanism has been optimized to enhance stability and comfort during wear, to reduce slipping during movement, and to better withstand vertical forces. Third, and most importantly, the device takes “guiding hip-dominant force application patterns during squats” as the core design goal, rather than merely providing support or generic resistance. Through reverse resistance applied at the knee, the proposed exoskeleton is designed to promote the activation of hip muscles and inhibit excessive knee loading, thereby actively shaping a safer, hip-driven squat pattern, which is a functionality that most existing similar systems do not provide.

Moreover, although many exoskeletons have been investigated in rehabilitation medicine and occupational protection, few studies have examined the direct application of exoskeleton systems in sports training, especially in guiding and optimizing force application patterns for complex movements like squats. Existing studies mainly target clinical populations or industrial tasks, with limited evidence in healthy athletes, beginners, or aging individuals performing resistance training. In practice, however, a training-oriented exoskeleton that can provide adjustable external resistance, biomechanical feedback, and hip-dominant guidance might be beneficial for a broader demographic: it could assist beginners with weak athletic ability in establishing correct movement patterns and reducing injury risk during the entry stage; support patients recovering from knee joint injuries in conducting progressive squat-based rehabilitation under controlled loading; and help middle-aged and elderly individuals safely perform lower limb strength training by tuning resistance to their physical capacity (Flor-Unda et al., 2025). Whether a single device can meet these diverse needs, and how its resistance should be tuned to avoid long-term over-reliance and to preserve autonomous control, remain open questions that require systematic biomechanical evaluation.

In response to these gaps, the present study independently developed a new training-oriented wearable knee joint exoskeleton to help users form a scientific and standardized lower limb force application pattern during squats through adjustable external resistance and biomechanical feedback. Specifically, this work seeks to (i) design a clutch-disc-based resistance module capable of precise torque adjustment and stable fixation; (ii) experimentally evaluate whether the exoskeleton can redistribute joint power toward a hip-dominant pattern and off-load the knee during squats; and (iii) preliminarily explore how such a device might be configured to support different user profiles while acknowledging the need to balance external support with intrinsic muscular activation and autonomous motor control. It is expected that this device will reduce adverse compensation during training, reduce the risk of knee joint injuries, and provide innovative technical support for practical sports training and injury prevention.

## 2 Methods

### 2.1 Exoskeleton design

The exoskeleton demands intelligence, convenience, and efficiency. Structurally, it must first provide sufficient resistance torque to counteract the dynamic torque generated by the human knee joint. The generated resistance torque must be adjustable to accommodate the varying needs of different wearers. High-intensity training applications impose stringent requirements on the structural integrity of the exoskeleton. Finally, it is essential to design a fastening mechanism that ensures a secure fit while enhancing the wearer’s comfort. Based on these design requirements, iterative design trials of the training-oriented wearable knee joint exoskeleton were conducted.

While the knee joint exoskeleton based on a damping rotating shaft can provide sufficient resistance torque and achieve the function of resistance training, it exhibits certain shortcomings. Specifically, there is a non-linear relationship between the tension of the adjustment nut and the output friction torque of the damping rotating shaft, and the range of the adjustment nut is limited. This results in difficulties in accurately regulating the output torque of the exoskeleton. Furthermore, the fastening components cannot withstand vertical forces, which can cause the exoskeleton to slip downward.

To address these issues, this article proposes a knee joint exoskeleton based on a clutch disc. A specially designed clutch disc replaces the damping rotating shaft, and upgrades are implemented in other structural components.

**FIGURE 1:**
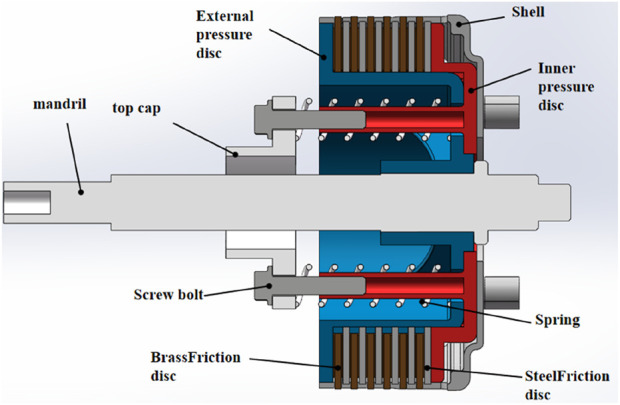
Improved clutch disc.

As shown in Figure 1, brass and steel friction discs are alternately mounted between the external pressure disc (blue) and the internal pressure disc (red). By adjusting the distance between the external and internal pressure discs, the pressure exerted on the brass and steel friction discs can be increased, resulting in greater friction torque that inhibits the rotation between the internal and external pressure discs. The four cylinders extending from the internal pressure disc are connected to the top cover via tightening bolts. Four springs are located between the top cover and the external pressure disc; tightening the bolts reduces the distance between the top cover and the internal pressure disc, causing the springs to exert a push force, which generates pressure between the internal and external pressure discs. When external forces are not applied to the system, the friction resistance between the inner and outer pressure discs is maximized; the tightening bolts can be adjusted to modify the maximum friction resistance.

When the rotating shaft moves to the left, the shoulder of the shaft pushes the external pressure disc. Due to the action of the springs, there is pressure between the internal and external pressure discs, causing the internal pressure disc, external pressure disc, brass friction discs, steel friction discs, tightening bolts, top cover, and springs to move synchronously to the left. At this point, an external force is applied to the left end face of the top cover, preventing it from moving to the left. Under the influence of this external force, the springs are compressed, separating the internal pressure disc from the external pressure disc and reducing the pressure between the brass and steel friction discs. This results in a decrease in the friction resistance between the internal and external pressure discs, thereby enabling stepless adjustment of the friction torque.

**FIGURE 2:**
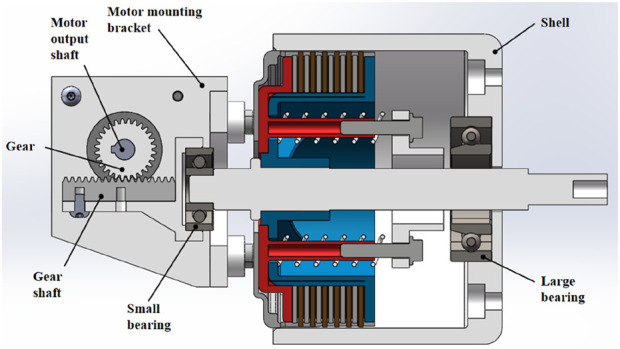
Clutch disc adjustment using motor control.

A system that uses a motor to adjust the clutch disc has been designed based on this principle, as illustrated in Figure 2. When the rotating shaft moves to the left, the shoulder of the shaft pushes the external pressure disc. The springs maintain the pressure between the internal and external pressure discs, resulting in the synchronous leftward movement of the internal pressure disc, the external pressure disc, the brass friction discs, the steel friction discs, the tightening bolts, the top cover, and the springs. An external force is then applied to the left end face of the top cover, hindering its leftward motion. As the external force is applied, the springs compress, separating the internal pressure disc from the external pressure disc and reducing the pressure between the brass and steel friction discs. This reduction in friction resistance between the internal and external pressure discs allows for precise, stepless adjustment of the friction torque. Based on this principle, a motor-driven clutch disc adjustment system was designed, as illustrated in Figure 2.

The outer casing is connected to the clutch disc mounting housing with bolts. The motor is securely attached to the mounting housing of the clutch disc through a motor mounting bracket, and the motor’s output shaft is connected to a gear that engages with a rack. Meanwhile, the motion trajectory of the rack is parallel to the axis of the rotating shaft.

When the motor rotates, it drives the rack to move horizontally to the right, which in turn pushes the rotating shaft to the right via a small bearing. As the top cover moves to the right over a certain distance, it engages with a large bearing, allowing the springs to compress and thereby meeting the operational requirements of the aforementioned clutch disc. The thigh link of the exoskeleton is rigidly connected to the outer casing, while the calf link is connected to the rotating shaft. The resistance torque at the knee joint can be adjusted by rotating the motor.

**FIGURE 3:**
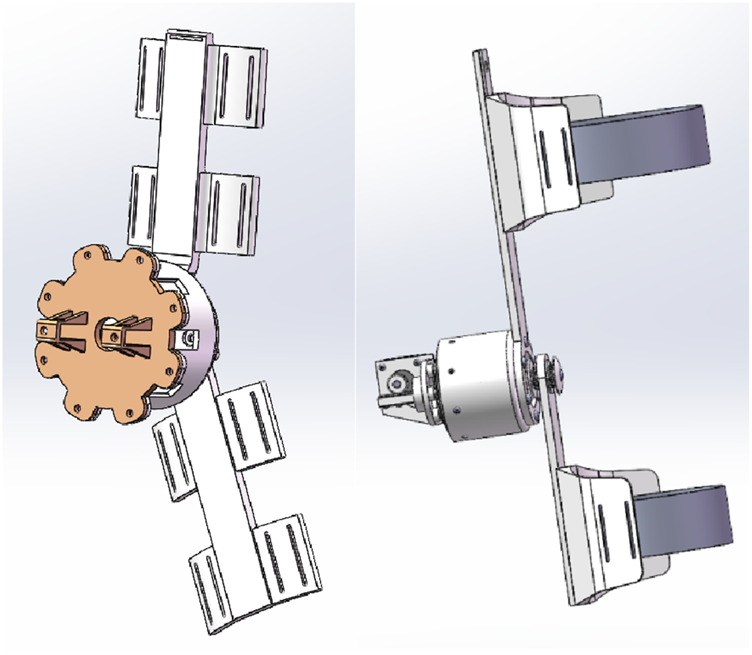
Clutch disc-based knee joint exoskeleton: manual adjustment (left) and motor-driven (right).

**FIGURE 4:**
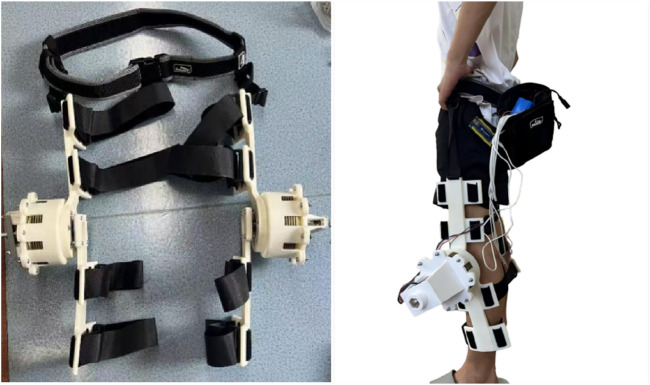
Prototype of the clutch disc-based knee joint exoskeleton: manual adjustment (left) and motor-driven (right).

**FIGURE 5:**
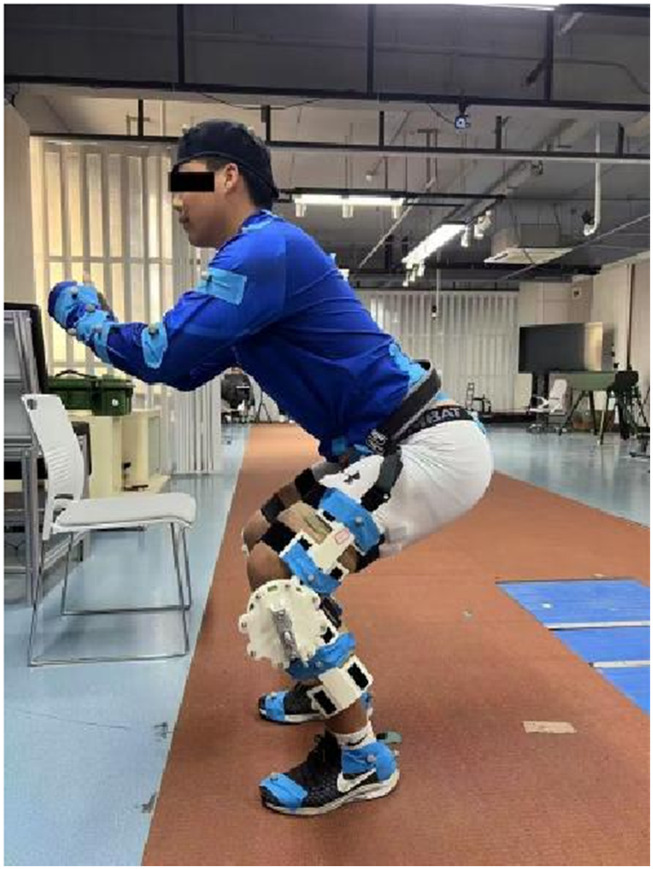
Schematic diagram of the experimental setup and subject posture.

Figure 3 presents two designed exoskeleton models: the left side illustrates the version for manual torque adjustment, while the right side showcases the motor-driven version. In the motor-driven version, a motor powers the rotating shaft, and the fastening components are designed with ergonomic considerations. The manual adjustment version drives shaft rotation by adjusting the length of a lead screw. The thigh link and calf link are integrated with fastening components, and certain weak structural areas have been reinforced to increase structural integrity. To improve the efficiency of the research and development process, the manual adjustment version of the exoskeleton design was used, with structural components produced through 3D printing. Necessary metal parts were contracted out to machining manufacturers for fabrication, resulting in the assembly of a prototype of the clutch disc-based knee joint exoskeleton, as shown in Figure 4. The posture of a participant wearing the exoskeleton during the study is shown in Figure 5.

Based on finite element analysis of the existing thigh and calf components made from resin materials, the applied forces have been set at 70 N, 140 N, and 210 N to ensure user safety as well as to evaluate the effectiveness of experimental group testing.

For the unilateral exoskeleton torque, the gravitational force GG is defined by the equation:

G=mg,

where G represents the gravitational force in Newtons (N), m is the mass in kilograms (kg), and g is the acceleration due to gravity, taken as 9.8 N/kg.

The calculation of mass m can be derived as follows:

$$G=70\;N\;g=9.8\;N/\text{kg}\;m=\frac{70}{9.8}\approx 7.14\text{ kg},$$

$$G=140\;N\;g=9.8\;N/\text{kg }m=\frac{140}{9.8}\approx 14.29\text{ kg},$$

$$G=210\;N\;g=9.8\;N\;\text{kg}/m=\frac{210}{9.8}\approx 21.43\text{ kg}.$$

Based on the measurement of the maximum distance from the fastening point on the support arm to the attachment point on the leg, which is 0.2 m, the torque ττ for the unilateral exoskeleton is defined by the equation:

τ=r×F,

where r is the distance (in meters) and F is the applied force (in Newtons). Accordingly, the torque settings for the unilateral exoskeleton are established as follows: 14 Nm, 28 Nm, and 42 Nm.

To ensure consistent mechanical load stimulation across the exoskeleton (torque-based) and control (barbell weight-based) groups, a two-step load matching protocol was implemented:

Biomechanical equivalence calculation: Load matching was based on the principle of equivalent mechanical work during the squat movement. The total mechanical work (W) performed by the lower limbs during squatting is determined by the product of force (F), displacement (s), and the cosine of the angle (θ) between force and displacement (W = F × s × cosθ). For the control group, the force is the gravitational force of the barbell (F_barbell = m_barbell × g), and the displacement is the vertical movement distance of the barbell during squatting (measured as the vertical displacement of the C7 marker, averaging 0.42 ± 0.03 m across participants). For the exoskeleton group, the torque (τ) applied at the knee joint is converted to equivalent force (F_exo = τ/r_knee), where r_knee is the effective moment arm of the knee joint (averaged 0.18 ± 0.02 m based on participant anthropometrics). The displacement corresponding to the knee torque is the angular displacement of the knee joint (converted to linear displacement of the lower leg endpoint: s_exo = θ_knee × r_knee, with θ_knee averaging 1.8 rad during squatting).

Verification and calibration: Prior to formal experiments, a pilot test was conducted with three additional healthy male participants (age: 21.0 ± 0.8 years, weight: 79.5 ± 3.2 kg, height: 178.3 ± 3.8 cm) to verify load equivalence. In the pilot experiment, the peak ground-reaction force (GRF) and total mechanical work were measured under two conditions: 28 Nm and 280 N. Results confirmed no significant differences in peak GRF (exoskeleton: 1,256 ± 89 N vs. control: 1,238 ± 94 N, p > 0.05) or total mechanical work (exoskeleton: 387 ± 42 J vs. control: 392 ± 38 J, p > 0.05) across matched loads, validating the load matching protocol.

### 2.2 Participants

The sample size was determined beforehand using G*Power (version 3.1.9.7) software. Based on a repeated measures analysis of variance (ANOVA) with a large effect size (f = 0.8), α = 0.05, and power = 0.8, a minimum of 10 participants was required (Li et al., 2025). A total of 15 healthy male participants were recruited, all from the Capital Institute of Physical Education. The participants had an average age of 20.4 ± 1.0 years, weight of 80.1 ± 4.7 kg, and height of 179.0 ± 4.5 cm. All participants reported no history of back pain or lower limb injuries. To avoid fatigue, the participants did not engage in vigorous exercise within the 24 h prior to the experiments. Each participant had prior experience with squat exercises and signed a written informed consent form before the experiment. This study was approved by the Ethics Committee of Capital Institute of Physical Education (2024A121).

### 2.3 Experimental tasks

Upon arrival at the laboratory, participants were first briefed on the entire experimental process and signed the consent form. Markers and electromyography (EMG) sensors were then attached to their bodies. Each participant underwent two experimental tests at least 30 min apart, which included the exoskeleton squat (exoskeleton condition) and barbell squat (control condition), to allow sufficient recovery time. A balanced randomization procedure was executed to determine whether the participant would wear the exoskeleton during the first or second experimental session. Before each experiment, participants were given at least 5 min to warm up. In each experiment, participants completed squat tasks at small, medium, and large loads. Under the exoskeleton condition, participants were required to perform three squat repetitions under torque conditions of 14 Nm (small load), 28 Nm (medium load), and 42 Nm (large load) applied to both knees in opposite directions. Under the control condition, the weights of the barbell were calculated based on the participants’ body morphology data, with participants performing three squat repetitions using a barbell of 14 kg (small load), 28 kg (medium load), and 43 kg (large load) to achieve a basic correspondence with the external loads used in the exoskeleton (because the designed exoskeleton is bilateral, the barbell weights are 7.14 kg × 2 ≈ 14 kg, 14.29 kg × 2 ≈ 29 kg, and 21.43 kg × 2 ≈ 43 kg). During each trial, participants stood with both feet on a force platform. They remained still for 3 s before squatting and after returning to an upright position to facilitate subsequent data recording. Overall, participants completed 18 squat trials (2 conditions × 3 loads × 3 trials).

### 2.4 Data collection and preprocessing

Experimental data were collected synchronously using a motion capture system, force platform system, and wireless surface EMG (sEMG) system. The OptiTrack 8-camera infrared motion capture system (NaturalPoint, United States) was used to collect the coordinate information of key points on the participants, with the conventional full-body 39-point model employed to select marker attachment locations. Two force platforms (Kistler, Switzerland) collected ground-reaction force (GRF) information from participants’ feet. sEMG signals were collected using a wireless EMG system (Delsys Trigno, United States) with a sampling frequency of 2,000 Hz.

#### 2.4.1 Data capture and initial processing

Motion capture data: Data were extracted using Motive software (version 3.0) accompanying the OptiTrack system, selecting a complete movement cycle from the last frame before the participant began to squat (onset defined as vertical GRF >10% of body weight) to the first frame of full standing (offset defined as knee joint angle <10° and vertical GRF returning to body weight ±5%). Marker trajectories were visually inspected to remove artifacts (e.g., marker occlusion and sudden position jumps). Missing data segments (<5 frames) were interpolated using cubic spline interpolation. A fourth-order Butterworth low-pass filter with a cutoff frequency of 6 Hz was applied to filter the coordinate data of key points to eliminate high-frequency noise.

GRF data: GRF signals were filtered using a fourth-order Butterworth low-pass filter with a cutoff frequency of 20 Hz. The center of pressure (COP) was calculated using built-in Kistler software, and abnormal GRF peaks (e.g., those exceeding three times body weight) were identified and excluded as motion artifacts.

sEMG data: A total of eight muscles on the right side of the body were tested, including the gluteus maximus (GM), biceps femoris (BF), semitendinosus (ST), rectus femoris (RF), vastus lateralis (VL), vastus medialis (VM), tibialis anterior (TA), and gastrocnemius (MG). EMG sensors were placed according to the Surface Electromyography for Non-Invasive Assessment of Muscles (SENIAM) guidelines, with skin prepared by shaving, abrasion, and cleaning with alcohol to reduce impedance (<5 kΩ). MATLAB 2019(a) was used for sEMG signal processing. First, a fourth-order Butterworth band-pass filter (20–450 Hz) was applied to remove low-frequency movement artifacts and high-frequency electrical noise. Then, a notch filter (50 Hz) was used to eliminate power line interference. Baseline correction was performed by subtracting the average signal amplitude during the 3-s static period before squatting. sEMG signals were full-wave rectified and smoothed using a moving average filter (window size: 50 ms) to obtain linear envelope signals. Artifact segments (e.g., signal amplitude >5 standard deviations of the baseline) were manually removed, and missing data were interpolated using linear interpolation.

#### 2.4.2 Inverse kinematics and dynamics analysis workflow

##### 2.4.2.1 Inverse kinematics (IK) in OpenSim

The filtered marker coordinate data were imported into OpenSim (version 4.4) and scaled to the full-body lumbar spine (FBLS) model based on participant anthropometrics (height, weight, thigh/calf length).

IK was performed using the OpenSim IK tool with a residual reduction algorithm, minimizing the difference between marker positions and model-generated marker positions (weighting factors: 1.0 for trunk and lower limb markers, 0.5 for upper limb markers). The IK solution was validated by ensuring residual errors <5 mm for all key markers (hip, knee, ankle, and sacrum).

Joint angle time series (hip, knee, and ankle in the sagittal plane) were extracted from the IK results, with a time step of 0.01 s.

##### 2.4.2.2 Inverse dynamics (ID) in OpenSim

For the exoskeleton condition, the knee joint torque (14 Nm, 28 Nm, and 42 Nm) was applied as an external torque in the direction opposite to knee flexion (negative sagittal plane torque) at the RKNE and LKNE markers of the FBLS model. The filtered GRF data were imported as external forces, with COP used to define the application point of GRF on the foot segment.

For the control condition, a constant vertical force (barbell weight × g) was applied at the C7 marker of the FBLS model, with the force direction aligned with gravity. Filtered GRF data were imported in a similar manner to the exoskeleton condition.

ID was performed using the OpenSim ID tool to calculate hip, knee, and ankle joint torques and powers, with joint power derived as the product of joint torque and angular velocity. All dynamic data were normalized to body weight (torque: Nm/kg, power: W/kg) to account for individual differences.

### 2.5 Result indicators and statistical analysis

As squats are a lower limb training action and a symmetrical movement, the analysis of kinematic and dynamic indicators focused on the right hip, knee, and ankle joints. For kinematic indicators, the joint range of motion and average joint angular velocity were selected for analysis.

Regarding dynamic indicators, because the external forces experienced by participants under the exoskeleton and control conditions were not precisely equal, it is not reasonable to directly compare torques between the two conditions. The purpose of this study is to analyze whether the exoskeleton improves the force application pattern, so the joint power contribution rate was selected for analysis. Specifically, in one trial, the average powers of the hip, knee, and ankle joints were compared to the sum of the average powers of all joints.

The muscle contribution rate was selected for analysis of electromyographic indicators, for the same reasons as for dynamic indicators. Specifically, in one trial, the integrated EMG (iEMG) of each muscle was compared to the sum of the iEMG of all muscles.

For all indicators, the Shapiro–Wilk test was used to assess normality; all data were found to follow a normal distribution. A 2 (conditions) × 3 (loads) repeated measures ANOVA was conducted, with the Bonferroni method applied for post-hoc testing. Effect sizes were quantified using partial eta squared (η^2^), with values of 0.01, 0.06, and 0.14 representing small, moderate, and large effects, respectively. The statistical significance was set at the 3-alpha level. Differences were considered statistically significant at p < 0.05, highly significant at p < 0.01, and very highly significant at p < 0.001.

## 3 Results

### 3.1 Joint range of motion

The range of motion of each joint and the results of repeated measures ANOVA are presented in Tables 1, 2, respectively. For the hip joint, neither the main effect of condition, nor the main effect of load, nor the condition × load interaction effect reached statistical significance. No significant differences were observed among all groups.

**TABLE 1:** Joint range of motion in the exoskeleton and control groups.

Joint	Group	Load	ROM (°)
Hip	EC	Low	111.68 ± 11.83
		Medium	110.01 ± 10.21
		High	106.45 ± 10.80
	CC	Low	109.78 ± 4.12
		Medium	112.77 ± 9.35
		High	113.99 ± 11.00
Knee	EC	Low	117.48 ± 5.81*
		Medium	120.72 ± 4.23*
		High	119.78 ± 8.64*
	CC	Low	125.66 ± 4.94
		Medium	128.27 ± 6.16
		High	127.96 ± 8.39
Ankle	EC	Low	25.05 ± 4.38*
		Medium	27.56 ± 2.91*
		High	26.81 ± 3.33*
	CC	Low	34.17 ± 4.23
		Medium	35.76 ± 3.93
		High	34.36 ± 3.66

EC denotes the exoskeleton condition. CC denotes the control condition. *, p< 0.05 with control condition.

**TABLE 2:** Mixed-model repeated measures ANOVA for joint range of motion.

Joint	Effect	F	p	η^2^
Hip	Condition	1.551	>0.05	0.052
	Load	0.121	>0.05	0.004
	Condition × load	1.955	>0.05	0.065
Knee	Condition	30.332	<0.01	0.523
	Load	1.714	>0.05	0.058
	Condition × load	0.024	>0.05	0.001
Ankle	Condition	116.285	<0.001	0.806
	Load	2.136	>0.05	0.103
	Condition × load	0.317	>0.05	0.011

With respect to the knee joint, no significant intergroup differences were detected; the main effect of condition was statistically significant, while the main effect of load and the condition × load interaction effect failed to reach significance. Further multiple comparison analyses demonstrated significant differences between the exoskeleton and control conditions across all three load levels (p < 0.05).

For the ankle joint, the main effect of the condition was statistically significant, whereas the main effect of load and the condition × load interaction effect were not significant. Subsequent multiple comparisons revealed significant differences between the exoskeleton and control conditions across all three load levels (p < 0.05).

The results for joint range of motion indicate that the exoskeleton decreases the range of motion at the knee and ankle joints.

### 3.2 Average joint angular velocity

The average angular velocities of each joint and the results of repeated measures ANOVA are presented in Tables 3, 4, respectively. For the hip joint, the main effect of condition, the main effect of load, and the condition × load interaction effect all reached statistical significance. Further multiple comparison analyses revealed significant differences between the exoskeleton and control conditions across all three load levels (p < 0.05). Specifically, in the exoskeleton condition, significant differences in average angular velocity were observed between the small/medium loads and the large load (p < 0.05); in the control condition, significant differences were detected between the small load and the medium/large loads (p < 0.05).

**TABLE 3:** Average joint angular velocity in the exoskeleton and control groups.

Joint	Group	Load	Angular velocity (°/s)
Hip	EC	Low	70.51 ± 11.82^*%^
		Medium	69.69 ± 13.50^*%^
		High	100.60 ± 12.08*
	CC	Low	92.66 ± 14.60^#%^
		Medium	118.07 ± 13.26
		High	125.16 ± 11.77
Knee	EC	Low	78.44 ± 7.32^*%^
		Medium	80.11 ± 8.25^*%^
		High	108.74 ± 16.94^*^
	CC	Low	105.45 ± 10.84^#%^
		Medium	132.84 ± 8.56
		High	141.07 ± 17.55
Ankle	EC	Low	15.00 ± 2.35^*^
		Medium	16.59 ± 2.74^*^
		High	18.09 ± 3.88^*^
	CC	Low	22.03 ± 5.03
		Medium	22.78 ± 3.50
		High	23.36 ± 3.45

EC denotes the exoskeleton condition. CC denotes the control condition. *, p < 0.05 with the control condition; %, p < 0.05 with a large load; #, p < 0.05 with a medium load.

**TABLE 4:** Mixed-model repeated measures ANOVA for average joint angular velocity.

Joint	Effect	F	p	η^2^
Hip	Condition	228.262	<0.001	0.891
	Load	37.401	<0.001	0.272
	Condition × load	7.912	<0.01	0.220
Knee	Condition	206.84	<0.001	0.881
	Load	54.243	<0.001	0.660
	Condition × load	9.158	<0.01	0.246
Ankle	Condition	63.18	<0.001	0.693
	Load	2.896	>0.05	0.094
	Condition × load	0.462	>0.05	0.016

For the knee joint, the main effect of condition, the main effect of load, and the condition × load interaction effect were all statistically significant. Further multiple comparisons demonstrated significant differences between the exoskeleton and control conditions across all three load levels (p < 0.05). In detail, in the exoskeleton condition, significant differences were found between the small/medium loads and the large load (p < 0.05); in the control condition, significant differences were identified between the small load and the medium/large loads (p < 0.05).

For the ankle joint, the main effect of condition was statistically significant, while neither the main effect of load nor the condition × load interaction effect reached significance. Further multiple comparisons revealed significant differences between the exoskeleton and control conditions across all three load levels (p < 0.05).

Collectively, the results on average joint angular velocity indicate that, under the exoskeleton condition, the average angular velocities of both the hip and knee joints were lower than those under the control condition, with significant performance discrepancies observed across different load levels. In contrast, although the average angular velocity of the ankle joint was significantly lower in the exoskeleton condition than in the control condition, the effect of load on the ankle joint was not statistically significant.

### 3.3 Joint power contribution rate

The power contribution rate of each joint and the results of repeated measures ANOVA are presented in Tables 5, 6, respectively. For the hip joint, the main effect of condition, the main effect of load, and the condition × load interaction effect all reached statistical significance. Further multiple comparison analyses demonstrated significant differences between the exoskeleton and control conditions across all three load levels (p < 0.05). Specifically, in the exoskeleton condition, the power contribution rate under the medium load was significantly different from that under the large load (p < 0.05).

**TABLE 5:** Joint power contribution rate in the exoskeleton and control groups.

Joint	Group	Load	Power contribution rate (%)
Hip	EC	Low	61.07 ± 7.29^*^
		Medium	30.67 ± 7.60^*%^
		High	8.40 ± 2.26^*^
	CC	Low	43.33 ± 5.35
		Medium	50.07 ± 5.28
		High	6.73 ± 1.79
Knee	EC	Low	63.80 ± 6.98^*%^
		Medium	28.80 ± 6.99^*%^
		High	7.47 ± 2.83^*^
	CC	Low	48.67 ± 6.40^#%^
		Medium	39.68 ± 5.34
		High	11.80 ± 2.73
Ankle	EC	Low	54.27 ± 4.28^*%^
		Medium	39.93 ± 4.37^*^
		High	6.13 ± 1.68^*^
	CC	Low	48.47 ± 9.51^#%^
		Medium	42.60 ± 8.15^%^
		High	8.93 ± 2.22

EC denotes the exoskeleton condition. CC denotes the control condition. *, p < 0.05 with control condition; %, p < 0.05 with a large load; #, p < 0.05 with a medium load.

**TABLE 6:** Mixed-model repeated measures ANOVA for the joint power contribution rate.

Joint	Effect	F	p	η^2^
Hip	Condition	79.493	<0.001	0.740
	Load	4.196	<0.05	0.140
	Condition × load	6.449	<0.05	0.187
Knee	Condition	66.893	<0.001	0.705
	Load	10.493	<0.01	0.273
	Condition × load	12.536	<0.01	0.309
Ankle	Condition	19.266	<0.01	0.408
	Load	7.306	<0.01	0.207
	Condition × load	12.265	<0.01	0.305

For the knee joint, the main effect of condition, the main effect of load, and the condition × load interaction effect were all statistically significant. Further multiple comparisons revealed significant differences between the exoskeleton and control conditions across all three load levels (p < 0.05). In the exoskeleton condition, significant differences were detected between the medium load and the large load, as well as between the small load and the large load (p < 0.05). In the control condition, significant differences were identified between the small load and both the medium and large loads (p < 0.05).

For the ankle joint, the main effect of condition, the main effect of load, and the condition × load interaction effect were all statistically significant. Further multiple comparisons demonstrated significant differences between the exoskeleton and control conditions across all three load levels (p < 0.05). In the exoskeleton condition, significant differences were observed between the small and large loads (p < 0.05); in the control condition, significant differences were observed across all three load levels (p < 0.05).

Collectively, the results about joint power contribution rate indicate that, under the exoskeleton condition, the power contribution rate of the hip joint was higher than that in the control condition. In contrast, the power contribution rate of the knee joint was lower than that in the control condition. Notably, each joint exhibited significant discrepancies in power contribution rate across different load levels. For the ankle joint, significant differences were also observed under the exoskeleton condition, yet the variation trends differed across different load levels.

### 3.4 Muscle contribution rate

#### 3.4.1 Gluteus maximus (GM)

**FIGURE 6:**
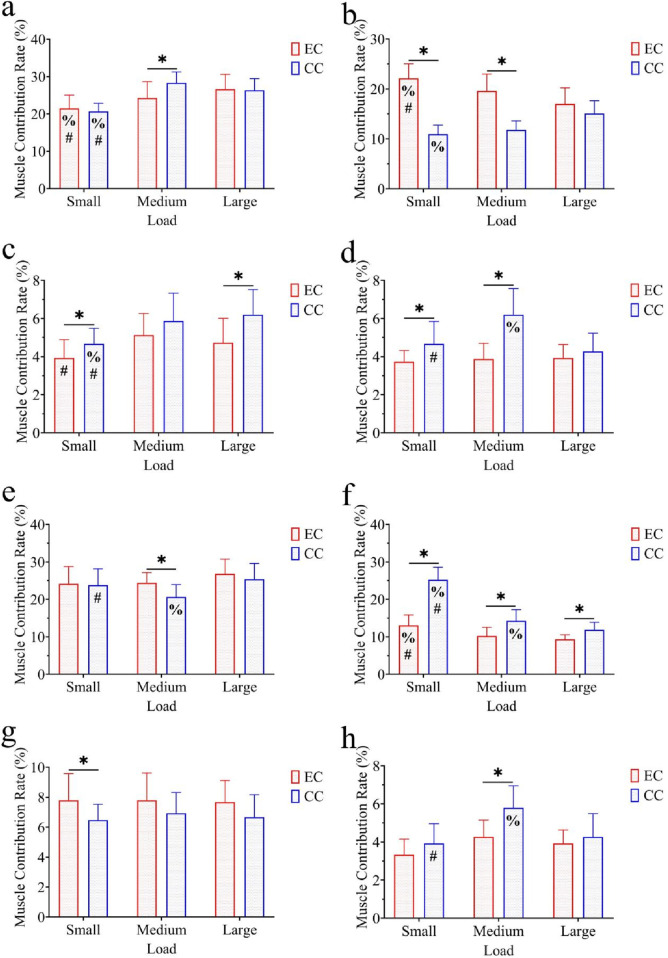
Comparison of muscle contribution rates: **(a)** gluteus maximus (GM), **(b)** biceps femoris (BF), **(c)** semitendinosus (ST), **(d)** rectus femoris (RF), **(e)** vastus lateralis (VL), **(f)** vastus medialis (VM), **(g)** tibialis anterior (TA), and **(h)** gastrocnemius (MG). EC denotes the exoskeleton condition, while CC denotes the control condition. The bars represent the standard deviation. *, p < 0.05 with control condition; %, p < 0.05 with large load; #, p < 0.05 with medium load.

Under the exoskeleton condition, the GM contribution rates for small, medium, and large load tasks were 21.47 ± 3.58, 24.27 ± 4.38, and 26.60 ± 4.01, respectively. Under the control condition, they were 20.67 ± 2.19, 28.33 ± 2.92, and 26.33 ± 3.13. The repeated measures ANOVA results showed that the main effect of condition was not significant (F = 1.574, p > 0.05, η^2^ = 0.053); the main effect of load was significant (F = 26.488, p < 0.001, η^2^ = 0.486), and the interaction effect of condition × load was significant (F = 5.003, p < 0.05, η^2^ = 0.152). Further multiple comparisons indicated significant differences between the exoskeleton and control conditions under the medium load (p < 0.05); in the exoskeleton condition, significant differences were found between small loads and medium/large loads (p < 0.05); in the control condition, significant differences were observed between small loads and medium/large loads (p < 0.05) (Figure 6a).

#### 3.4.2 Biceps femoris (BF)

Under the exoskeleton condition, the BF contribution rates for small, medium, and large load tasks were 22.13 ± 2.90, 19.60 ± 3.38, and 17.00 ± 3.21, respectively. Under the control condition, they were 10.93 ± 1.83, 11.80 ± 1.82, and 15.07 ± 2.58. The repeated measures ANOVA results revealed a significant main effect of condition (F = 182.103, p < 0.001, η^2^ = 0.867); the main effect of load was not significant (F = 0.673, p > 0.05, η^2^ = 0.023), while the interaction effect of condition × load was significant (F = 21.016, p < 0.001, η^2^ = 0.429). Further multiple comparisons showed significant differences between the exoskeleton and control conditions for small/medium loads (p < 0.05); in the exoskeleton condition, significant differences were found between small loads and medium/large loads (p < 0.05); in the control condition, significant differences were observed between small loads and large loads (p < 0.05) (Figure 6b).

#### 3.4.3 Semitendinosus (ST)

Under the exoskeleton condition, the ST contribution rates for small, medium, and large load tasks were 3.93 ± 0.96, 5.13 ± 1.13, and 4.73 ± 1.28, respectively. Under the control condition, they were 4.67 ± 0.82, 5.87 ± 1.46, and 6.20 ± 1.32. The repeated measures ANOVA results revealed a significant main effect of condition (F = 11.200, p < 0.01 η^2^ = 0.286), a significant main effect of load (F = 12.397, p < 0.001, η^2^ = 0.307), while the interaction effect of condition × load was not significant (F = 1.119, p > 0.05, η^2^ = 0.041). Further multiple comparisons found significant differences between small and large loads for the exoskeleton and control conditions (p < 0.05); in the exoskeleton condition, significant differences were observed between small and medium loads (p < 0.05); in the control condition, significant differences were found between small loads and medium/large loads (p < 0.05) (Figure 6c).

#### 3.4.4 Rectus femoris (RF)

Under the exoskeleton condition, the RF contribution rates for small, medium, and large load tasks were 3.73 ± 0.59, 3.87 ± 0.83, and 3.93 ± 0.70, respectively. Under the control condition, they were 4.67 ± 1.18, 6.20 ± 1.37, and 4.20 ± 0.96. The repeated measures ANOVA results showed a significant main effect of condition (F = 24.862, p < 0.001, η^2^ = 0.470), a significant main effect of load (F = 10.077, p < 0.001, η^2^ = 0.265), and a significant interaction effect of condition × load (F = 10.106, p < 0.001, η^2^ = 0.265). Further multiple comparisons indicated significant differences between the exoskeleton and control conditions for small and medium loads (p < 0.05); in the control condition, significant differences were observed between medium loads and small/large loads (p < 0.05) (Figure 6d).

#### 3.4.5 Vastus lateralis (VL)

Under the exoskeleton condition, the VL contribution rates for small, medium, and large load tasks were 24.13 ± 4.64, 24.40 ± 2.75, and 26.80 ± 3.95, respectively. Under the control condition, they were 23.80 ± 4.36, 20.60 ± 3.36, and 25.40 ± 4.19. The repeated measures ANOVA results indicated a significant main effect of condition (F = 4.872, p < 0.05, η^2^ = 0.148), a significant main effect of load (F = 6.436, p < 0.01, η^2^ = 0.318), and the interaction effect of condition × load was not significant (F = 1.548, p > 0.05, η^2^ = 0.052). Further multiple comparisons revealed significant differences between the exoskeleton and control conditions under medium load (p < 0.05); in the control condition, significant differences were found between medium loads and small/large loads (p < 0.05) (Figure 6e).

#### 3.4.6 Vastus medialis (VM)

Under the exoskeleton condition, the VM contribution rates for small, medium, and large load tasks were 13.07 ± 2.76, 10.20 ± 2.34, and 9.33 ± 1.23, respectively. Under the control condition, they were 25.20 ± 3.38, 14.27 ± 2.99, and 11.87 ± 2.00. The repeated measures ANOVA results indicated a significant main effect of condition (F = 129.082, p < 0.001, η^2^ = 0.822), a significant main effect of load (F = 96.974, p < 0.001, η^2^ = 0.776), and a significant interaction effect of condition × load (F = 31.430, p < 0.001, η^2^ = 0.529). Further multiple comparisons revealed significant differences between the exoskeleton and control conditions across all three loads (p < 0.05); in the exoskeleton condition, significant differences were found between small loads and medium/large loads (p < 0.05); in the control condition, significant differences were observed between all three loads (p < 0.05) (Figure 6f).

#### 3.4.7 Tibialis anterior (TA)

Under the exoskeleton condition, the TA contribution rates for small, medium, and large load tasks were 7.80 ± 1.78, 7.80 ± 1.82, and 7.67 ± 1.45, respectively. Under the control condition, they were 6.47 ± 1.06, 6.93 ± 1.39, and 6.67 ± 1.50. The repeated measures ANOVA results indicated a significant main effect of condition (F = 9.355, p < 0.01, η^2^ = 0.250); however, the main effect of load was not significant (F = 0.228, p > 0.05, η^2^ = 0.008), and the interaction effect of condition × load was not significant (F = 0.207, p > 0.05, η^2^ = 0.007). Further multiple comparisons indicated significant differences between the exoskeleton and control conditions under small loads (p < 0.05) (Figure 6g).

#### 3.4.8 Gastrocnemius (MG)

Under the exoskeleton condition, the MG contribution rates for small, medium, and large load tasks were 3.33 ± 0.82, 4.27 ± 0.88, and 3.93 ± 0.70, respectively. Under the control condition, they were 3.93 ± 1.03, 5.80 ± 1.15, and 4.27 ± 1.22. The repeated measures ANOVA results indicated a significant main effect of condition (F = 14.904, p < 0.01, η^2^ = 0.347), a significant main effect of load (F = 16.141, p < 0.01, η^2^ = 0.366), while the interaction effect of condition × load was not significant (F = 3.153, p > 0.05, η^2^ = 0.101). Further multiple comparisons revealed significant differences between the exoskeleton and control conditions under medium load (p < 0.05); in the control condition, significant differences were found between medium loads and small/large loads (p < 0.05) (Figure 6h).

All muscles showed differences between the two conditions, indicating that the participants adopted different muscle activation strategies under the two conditions. Under low load, the contribution rates of the BF and TA in the exoskeleton condition were higher than those in the control condition, while the contribution rates of the ST, RF, and VM were lower than those in the control condition. Under medium load, the contribution rates of the BF and VL in the exoskeleton condition were higher than those in the control condition, while the contribution rates of the GM, ST, RF, VM, and MG were lower than those in the control condition. Under high loads, the contribution rates of the ST and VM in the exoskeleton condition were lower than those in the control condition.

## 4 Discussion

### 4.1 Impact of exoskeleton on joint kinematic parameters

This study explored the application of a resistance-type knee exoskeleton in squat tasks, focusing on changes in joint range of motion and average angular velocity as kinematic indicators. Participants under the exoskeleton condition exhibited a smaller joint range of motion and significantly lower average joint angular velocities (hip: η^2^ = 0.891; knee: η^2^ = 0.881; ankle: η^2^ = 0.693), all representing large effect sizes. These findings provide important insights into the advantages and limitations of using exoskeletons, particularly regarding their long-term effects on movement function.

First, the advantages of the exoskeleton in hip and knee joint motion control should not be overlooked. Joint ranges of motion were significantly lower under the exoskeleton condition than under the control condition, particularly inhibiting the forward shift of the knee and ankle joints (η^2^ = 0.523 for knee, η^2^ = 0.806 for ankle, both large effects). By applying a reverse resistance, the exoskeleton prompted participants to adopt a hip-dominant force application pattern, effectively reducing the load on the knee joint during squats. This hip-dominant strategy not only increases the biomechanical safety of the movements and significantly decreases the risk of sports injuries but may also improve the efficiency of muscle use. Research shows that maintaining proper hip flexion and posterior displacement is key to optimizing force lines and improving power output (Baumgart et al., 2021).

Moreover, the exoskeleton’s ability to reduce participants’ movement speed indicates that the execution of movements becomes more stable and controlled. Compared to rapid, sudden movements, slower and controlled movement velocities can promote the standardization of technique, thereby supporting trainees in developing a more scientific and effective squat technique (Gentil et al., 2007). This process helps optimize participants’ biomechanical performance during exercise and may even enhance adaptive neural connections in the brain, facilitating motor learning and the formation of long-term memory (Levin et al., 2015).

However, the use of the exoskeleton also has its limitations. Although it improves the standardization and safety of movements, it also restricts the natural range of joint motion. The significant reduction in the range of motion at the knee and ankle joints under the exoskeleton condition may impact participants’ freedom of movement and flexibility. From a long-term perspective, such constraints may gradually reduce athletes’ flexibility, as prolonged limited joint mobility can lead to adaptive changes in connective tissue and muscle-tendon units, reducing extensibility. For athletes requiring a broad range of motion (e.g., gymnasts and dancers) or whose performance relies on flexible lower limb movements, this limitation may hinder technical progression. For example, reduced ankle dorsiflexion range may affect squat depth adaptation during high-intensity training, while limited knee mobility may compromise dynamic stability in multi-directional movements. This restriction could lead to poor load distribution in certain movements, thereby affecting the overall engagement and effectiveness of the muscle activation paths. Studies have indicated that excessive constraints on joint motion may lead to decreased muscle adaptability and functional impairment (Schoenfeld and Grgic, 2020).

In summary, the resistance-type exoskeleton demonstrates advantages in squat training by increasing movement standardization (large effect sizes for angular velocity reduction), reducing knee joint load, and optimizing power output patterns. However, this device may also limit joint range of motion (large effects on the knee and ankle), resulting in decreased flexibility and hindering long-term technical development with prolonged use. Therefore, in training and rehabilitation applications, it is essential to flexibly adjust the settings of the exoskeleton based on individual needs and goals, combining it with targeted mobility training (e.g., ankle dorsiflexion stretches, knee flexion range exercises) to mitigate long-term flexibility impairments and achieve optimal training outcomes.

### 4.2 Impact of exoskeleton on joint force application patterns

In squat training, the choice of primary joints for force application has profound implications for exercise performance and joint health. Research indicates that a hip-dominant force application pattern offers several significant advantages over a knee-dominant pattern, with the exoskeleton further optimizing these benefits while requiring attention to long-term muscle adaptability.

First, hip-dominant force application effectively increases power output during squats. The hip joint is the primary source of force generation, especially during high-load exercises. When athletes rely on the hip joint for force application, they can engage large muscle groups, such as the gluteus maximus and hamstrings, thereby increasing force output (Dewald et al., 2023). This pattern allows athletes to control the movement process more effectively, reduce fatigue, and lower the risk of injury. In contrast, a knee-dominant force application often places excessive load on the knee joint, increasing the risk of injuries such as ligament damage or patellar injuries (Hewett et al., 2006). The exoskeleton significantly increases hip joint power contribution (η^2^ = 0.740, large effect) while reducing knee joint contribution (η^2^ = 0.705, large effect), confirming optimized power output distribution.

Second, the hip joint helps maintain the body’s center of gravity during squats, ensuring a more balanced mechanical load throughout the movement. In knee-dominant force applications, athletes are often prone to excessive forward movement of the knees, resulting in a forward lean that increases stress on the knee joint and may lead to technical errors. Conversely, the hip-dominant approach promotes better posture and stability, facilitating safer movement execution (Zawadka et al., 2020). The exoskeleton’s constraint effect (reflected by reduced joint range of motion and angular velocity) reinforces this stable posture, with a large effect size (η^2^ = 0.891 for hip angular velocity) indicating strong intervention effectiveness.

The hip-dominant force application helps improve exercise economy. Exercise economy refers to the ratio of energy expenditure to force output during specific movements. Studies indicate that athletes engaging in hip-dominant force application during squats can generate more power with less energy expenditure. This efficient use of energy enhances athletic performance and helps athletes maintain stamina over longer training or competition periods (Fuglsang et al., 2017).

However, long-term use of the exoskeleton may impact muscle adaptability and autonomous force control. The significant power redistribution (hip contribution increased by 20%–40%; knee contribution decreased by 20%–30%) indicates that muscles adapt to the exoskeleton-modified load distribution. Prolonged reliance may reduce the adaptability of knee-related muscles (e.g., vastus medialis) to high loads and compromise athletes’ ability to independently adjust force application patterns when the exoskeleton is not used. For example, athletes accustomed to hip-dominant movement under exoskeleton support may struggle to maintain balanced force distribution between hip and knee joints during unassisted squats, affecting movement efficiency and increasing injury risk. This is particularly critical for competitive athletes, as autonomous force control and muscle adaptive capacity are core components of athletic performance.

In this context, the application of the exoskeleton further optimizes the effectiveness of hip-dominant force application. The exoskeleton provides additional support, helping athletes maintain the correct force application pattern under various load conditions. Research findings show that participants using the exoskeleton exhibited a significant increase in hip joint power contribution during squats, while the load on the knee joint was relatively reduced, achieving optimization of power output (Kim et al., 2015). This feature indicates that exoskeleton-assisted squats both increase the biomechanical efficiency of power exercise and improve the safety of the movements (Ishida et al., 2022).

In summary, the hip-dominant force application pattern exhibits advantages over the knee-dominant pattern in power output, movement stability, and economy. The use of the exoskeleton further increases these effects (supported by large effect sizes for power redistribution), making exoskeleton-assisted squats more effective and safer than traditional barbell squats. However, long-term use may reduce muscle adaptability and autonomous force control capabilities, requiring rational use of the device combined with unassisted training to balance safety and functional development.

### 4.3 Advantages and limitations of exoskeleton on muscle activation

Under the exoskeleton condition, participants’ hip joint power contributions significantly increased, indicating that the exoskeleton enabled better utilization of major muscle groups such as the GM and BF(BF contribution rate η^2^ = 0.867, large effect), thereby increasing power output markedly. Particularly during small and medium load tasks, the contribution rates of the BF were significantly higher than those observed in the control conditions. This force application pattern increases exercise performance. It effectively reduces the load on the knee joint, as the contributions of knee joint muscles (such as the VM and RF) were lower (VM η^2^ = 0.822, large effect), thus alleviating pressure on the knee and reducing injury risk (Crossley et al., 2011). By optimizing suitable force application patterns, athletes can effectively reduce joint loads during dynamic movements, consequently improving performance and safety. The support and stability provided by the exoskeleton enable participants to maintain better posture during squats, thereby minimizing the risk of injury due to technical errors. Furthermore, the use of the exoskeleton may improve overall biomechanical efficiency of movement by promoting activation of specific muscle groups, particularly under lighter loads.

However, there are certain limitations to the external skeleton condition. Although the contribution of the GM did not increase significantly under medium loads, this suggests that athletes may not activate all relevant muscle groups sufficiently when relying on the exoskeleton, leading to insufficient activation of certain muscles. Additionally, participants may find it challenging to replicate the same force application patterns without the exoskeleton, which could impact the transfer of training effectiveness. More importantly, this reflects potential dependency issues. Prolonged exoskeleton use may lead athletes to form movement habits dependent on external support, making it difficult to independently maintain correct force application patterns without the device. This dependency can reduce training transfer effectiveness, as the movement patterns and muscle activation strategies developed under exoskeleton support may not be effectively applied to unassisted training or competition scenarios. During the initial adaptation period, some users may also experience fluctuations in movement control stability, further increasing the risk of dependency if not properly guided. Some users may take time to adapt to using the exoskeleton, leading to fluctuations in movement control stability during the initial stages.

The measurement system has inherent limitations that should be acknowledged. The study used the OptiTrack 8-camera motion capture system, Kistler force platforms, and Delsys Trigno wireless EMG system. The motion capture system has a spatial resolution of ∼0.1 mm and a sampling frequency of 100 Hz, which meets basic kinematic measurement needs but may not capture ultra-fast movement details (e.g., instantaneous joint angular velocity changes during explosive squats). The force platforms have a measurement range of 0–20 kN and accuracy of ±0.1% F.S., but ground-reaction force (GRF) measurement may be affected by foot placement stability: small shifts in foot position during squats can introduce measurement errors. For the EMG system (sampling frequency 2000 Hz, filter range 20–450 Hz), skin–electrode contact quality and muscle crosstalk may influence signal accuracy, especially for deep muscles or adjacent muscle groups (e.g., semitendinosus and biceps femoris). Additionally, the measurement process is constrained by laboratory conditions: participant movement may be affected by the wearable devices, leading to differences from real-world training scenarios. These limitations should be considered when interpreting results, as they may impact the validity and generalizability of the observed muscle activation patterns.

In the control condition, all relevant muscle groups (including the VM, RF, and ST) could be fully utilized, leading to coordinated activation of all muscle groups. This approach enhances dynamic coordination and comprehensive technical training. Additionally, without the exoskeleton, the activation levels of the RF and VM significantly increased, promoting overall strength and endurance (Fuglsang et al., 2017). In addition, in terms of self-regulating strength control abilities and motor skills, participants in the control condition could better adapt and develop skills fundamental for various daily activities and professional sports.

However, the control condition also has its limitations. Without the support of the exoskeleton, the knee joint bears heavier pressure, especially under high loads, increasing the risk of injury, such as ligament damage and patellar injuries. Studies have shown that improper squat form can lead to excessive stress on the knee joint; thus, correct force application is crucial for injury prevention (Sharp and Brouwer, 1997). Moreover, in the absence of external device support, athletes face greater challenges maintaining proper posture, increasing the risk of technical errors under high loads. The efficiency of force application may be lower, as the activation levels of some muscle groups decrease under heavier loads, causing athletes to fatigue more easily than when using an exoskeleton.

In conclusion, both the exoskeleton and control conditions have their strengths. The exoskeleton condition allows for more effective application of hip-dominant force application, reduces knee joint burden, and enhances movement safety, but it may lead to imbalances in muscle contributions and dependency issues and is subject to measurement system constraints. In the control condition, athletes can coordinate the activation of all muscle groups, enhancing the autonomy of strength control but also increasing pressure on the knee joint and risk of technical errors. To maximize training effectiveness, the exoskeleton should be used as a temporary guidance tool rather than a long-term substitute, with gradual reduction of resistance and increased unassisted training to mitigate dependency and muscle imbalance risks.

### 4.4 Practical guidance

Short-term/rehabilitation: The exoskeleton offers a safe, coach-independent tool to engrain correct mechanics and prevent knee injury. For patients recovering from knee joint injuries or beginners learning squat techniques, it provides stable support to establish hip-dominant movement patterns without excessive knee stress.

Long-term/performance: Resistance should be progressively tapered and complemented with full-ROM free squats and targeted activation drills to preserve joint mobility and ensure training transfer. For elite athletes, the exoskeleton should be used selectively (e.g., only during technique correction phases) to avoid compromising muscle adaptability and autonomous force control.

For users with distinct anthropometric characteristics (e.g., shorter lower limbs and larger thigh circumferences), the following configuration steps are recommended: First, adjust the telescopic links to align the exoskeleton’s rotation axis with the user’s knee joint center (verified via motion capture marker calibration). Second, optimize the fastening strap tension to minimize relative slippage (targeting skin–exoskeleton interface pressure <30 kPa, measured via pressure sensors).

Overall, the device successfully bridges technology and exercise science by converting a high-risk movement into a safer, standards-compliant training modality. Individualized programming, adjunct exercises, and rational use of the device (avoiding long-term over-reliance) are recommended to optimize both safety and functional outcomes.

## 5 Conclusion

This study introduced a novel clutch-disc-based resistance knee exoskeleton and evaluated its immediate biomechanical effects during squat training. The findings indicate that the device effectively shaped movement technique by reducing peak angular velocities at the hip, knee, and ankle, thereby promoting slower and more controlled squats. A consistent hip-dominant load redistribution pattern was observed, with hip joint power contribution increasing by 20%–40% and knee joint contribution decreasing by 20%–30%, substantially unloading the patellofemoral and cruciate ligament structures. Nevertheless, trade-offs emerged, as muscle activation of the vastus medialis, rectus femoris, and tibialis anterior declined by up to 50%, suggesting potential deficits in neuromuscular coordination if the device is used in isolation.

## Data Availability

The raw data supporting the conclusions of this article will be made available by the authors, without undue reservation.
